# Letter to the editor regarding ‘Cognitive behavioral interventions for depression and anxiety in adults with neurological disorders: a systematic review and meta-analysis’

**DOI:** 10.1017/S0033291724003180

**Published:** 2025-02-04

**Authors:** Yongjia Zhou, Qingyong Zheng, Jinhui Tian

**Affiliations:** 1 Evidence-Based Medicine Center, School of Basic Medical Sciences, Lanzhou University, Lanzhou City, Gansu Province, China; 2 School of Nursing, Gansu University of Chinese Medicine, Lanzhou City, Gansu Province, China

**Keywords:** anxiety, cognitive behavioral therapy, depression, neurological disease, neuropsychiatry, psychotherapy

We recently read with great interest the article by Gandy M et al. in Psychological Medicine, titled ‘Cognitive behavioral interventions for depression and anxiety in adults with neurological disorders: a systematic review and meta-analysis’ (Gandy et al., 2024). This comprehensive review offers valuable insights into the efficacy of cognitive and behavioral interventions for alleviating symptoms of depression and anxiety across various neurological disorders (NDs). We commend the authors for their thorough analysis and synthesis of data from numerous randomized controlled trials (RCTs) in this critical area of neuropsychiatry. However, we believe several methodological concerns and interpretation issues warrant further discussion, which may clarify the study’s implications and enhance its applicability to clinical practice.

**Figure 1.**
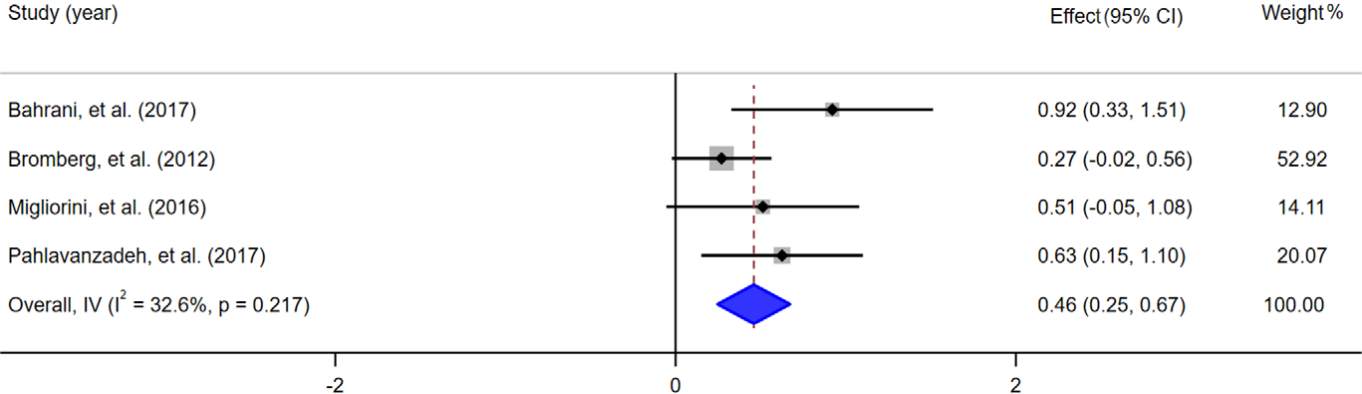
Forest plot of effects of interventions on anxiety under a fixed-effects model (DASS-A scale).

First, the authors directly opted for a random-effects model for the meta-analysis, which may have led to an overly optimistic estimation of the study result (Deeks et al., 2023). For example, the authors reported in the original article that the anxiety effect sizes varied when different anxiety scales were used, with the DASS-A scale showing a moderate effect (*g* = 0.52, 95% CI 0.23–0.79). However, when we converted the random-effects model to a fixed-effect model, the effect size was small (*g* = 0.46, 95% CI 0.25–0.67) (Figure 1). Therefore, we believe that the choice of an appropriate analysis model based on the degree of heterogeneity between studies would likely yield a more accurate representation of the combined evidence.

Second, a key assumption of the standardized mean difference (SMD) method is that the outcome measures used in different studies can be considered as linear transformations or incomplete measurements of the same construct (Murad et al., 2019). However, in this study, significant variability exists in the measurement tools used across the included studies to assess the same outcomes (e.g. HADS-D, DASS21-D, DASS42-D, BDI-II, and PHQ-9), making it difficult to satisfy this assumption. For example, the BDI-II may be more biased toward self-perceived emotional changes, while the HADS-D focuses more on the depressive symptoms of physically ill patients (Wu et al., 2021). Even with the use of SMD, these differences may not be fully eliminated, thereby reducing the credibility of the results. Authors and the broader readership should be aware of the potential impact brought about by this limitation and seek to optimize it in future research, similar to how they acknowledged the potential bias introduced by sample attrition.

Furthermore, the differences in intervention effects across control groups (e.g. treatment as usual, waiting list control, education) were substantial. When inactive control conditions (such as waiting list control) were used, studies tend to overestimate the true effect of the intervention (cognitive and behavioral interventions) group (Karlsson & Bergmark, 2015). Therefore, to better understand the influence of different control conditions, we suggest conducting a network meta-analysis in the future, as it can help reveal the true differences in the effects of various interventions through the construction of an evidence network and indirect comparisons (Ioannidis, 2009).

Finally, given the aforementioned concerns, along with the biases and limitations discussed in the study, we believe the authors’ interpretation of the effects of cognitive and behavioral interventions on depression and anxiety in patients with various NDs based solely on data differences may be premature. The Grade of Recommendations Assessment, Development, and Evaluation (GRADE) system, which provides a systematic assessment of the quality of evidence and the strength of recommendations, could better help readers understand the strengths and limitations of the evidence (Guyatt et al., 2008). For example, the authors concluded that cognitive and behavioral interventions showed small-to-moderate anti-depressive effects in ND patients, but when we conducted the GRADE assessment, the evidence was classified as very low (Table 1), and the authors should be more cautious in their interpretation of the results.

**Table 1.** GRADE evidence—depression symptoms at post-intervention

Certainty assessment							Effect	Certainty	Importance
No of studies	Study design	Risk of bias	Inconsistency	Indirectness	Imprecision	Other considerations	Hedges’s g (95% CI)		
57	Randomized trials	Not serious	Serious^a^	Serious^b^	Serious^c^	Publication bias (Egger’s test *p* = 0.03)	0.45 (0.35, 0.54)	⨁ ◯ ◯ ◯ very low	Important

CI: Confidence interval; Hedges’s *g*: effect size.

a There is substantial heterogeneity in pooled results.

b Variation in population type and contents of psychological interventions.

c More than half had sample size less than 50 per group.

All in all, we would like to express our gratitude to the authors for their exploration and contributions to this field. We believe that addressing the aforementioned limitations will enhance the quality of research but also promote the optimization of cognitive and behavioral interventions in clinical applications, ultimately benefiting patients with NDs who experience anxiety and depression.
